# Primary care quality between Traditional Tibetan Medicine and Western Medicine Hospitals: a pilot assessment in Tibet

**DOI:** 10.1186/s12939-015-0174-y

**Published:** 2015-05-14

**Authors:** Wenhua Wang, Leiyu Shi, Aitian Yin, Zongfu Mao, Elizabeth Maitland, Stephen Nicholas, Xiaoyun Liu

**Affiliations:** School of Public Health, Wuhan University, 115 Donghu Road, Wuhan, Hubei Province 430071 People’s Republic of China; Center for Health Management and Policy, Shandong University, 44 Wenhuaxilu, Jinan, Shandong Province 250012 People’s Republic of China; Johns Hopkins Bloomberg School of Public Health, Johns Hopkins Primary Care Policy Center, 624 North Broadway, Baltimore, MD 21205 USA; University of Newcastle, Newcastle, NSW 2308 Australia; School of Management, Australian School of Business, University of New South Wales, Sydney, NSW 2052 Australia; School of Management, Tianjin Normal University, West Bin Shui Avenue, Tianjin, 300074 People’s Republic of China; School of International Business, Beijing Foreign Studies University, 19 North Xisanhuan Avenue, Haidian Beijing, 100089 People’s Republic of China; Guangdong Research Institute for International Strategies, Guangdong University of Foreign Studies, 2 Baiyun North Avenue, Baiyun, Guangzhou, Guangdong 510420 People’s Republic of China; China Center for Health Development Studies, Peking University, 38 Xueyuan Road, Haidian District Beijing, 100191 People’s Republic of China

**Keywords:** Primary care, Primary care assessment tool, Traditional Tibetan medicine, Western medicine

## Abstract

**Introduction:**

This paper assesses both patients’ perspectives on the differences in primary care quality between traditional Tibetan medicine (TTM) hospitals and western medicine (WM) hospitals and the efficacy of the government’s investment in these two Prefecture-level primary care structures in Tibet.

**Method:**

A validated Tibetan version of the Primary Care Assessment Tool (PCAT-T) was used to collect data on 692 patients aged over 18 years old, who reported the sampling site was their regular source of health care. T-tests were performed to compare the separate and total primary care attributes between WM hospitals and TTM hospitals. Multiple linear regression analysis was conducted to examine the association of the health care setting with primary care attributes while controlling for socio-demographic, health service use and health status characteristics.

**Results:**

Compared to WM hospitals, the results showed that TTM hospitals had patients who were older (15.8 % versus 8.4 % over 60 years); with lower education levels (66.0 % versus 35.8 % with below junior high school ) and income levels (46.9 % versus 26.5 % with annual household income below 30,000RMB); more likely to be married (79.2 % versus 60.5 %); made less frequent health care visits; and had higher self-rated health status. Overall, patients assessed the primary care performance in TTM hospitals significantly higher (80.0) than WM hospitals (74.63). There were no differences in health care assessment by patient gender, age, income, education, marital status and occupation.

**Conclusions:**

TTM patients reported better primary care experiences than patients using WM hospitals, which validated the government’s investment in traditional Tibetan medicine.

## Introduction

Considerable evidence has shown that countries with a strong primary care-led health system have a better, and a more equitable distribution, of population health outcomes, and achieve these at a lower cost than countries with weak primary care health systems [1–3]. There is also clear evidence that health system performance is enhanced by good primary care service delivery [4]. Good primary health care is assessed against first contact, longitudinality, comprehensiveness and coordination [1]. In Tibet, the health system is a primary care based system, comprising both primary care clinics and outpatient departments of hospitals. During the past six decades, the Tibet health system has improved significantly the local population’s health, with the maternal mortality rate falling from 5000/100,000 to 154.51/100,000, the infant mortality rate falling from 430 ‰ to 19.97 ‰ and life expectancy increasing from 35.5 years to 67 years [5].

But the health system in China, including Tibet, has faced widespread public discontent stemming from constrained access to health care, its affordability, especially financial risks associated with out-of-pocket medical expenses, and growing inequalities in access to health care across regions, for different socioeconomic groups and between urban and rural populations [6]. In response, China unveiled an ambitious health-care reform program in 2009, including improvements to the primary health care delivery system, reforms in western and traditional medicine public hospitals and special measures to improve the primary health care system. One aim of the 2009 reforms was to boost the gatekeeping function of primary health care, as a filter for allocating patients to further specialist and hospital care. Given Tibet’s rural bias and relatively poor socioeconomic population, the health reforms sought to guarantee wide geographical coverage and unrestricted access to a physician at prefecture hospitals (PH), county hospitals (CH) and township health centers (THC) [7, 8]. At the PH level, the Tibetan system involves both western and traditional Tibetan medicine hospitals.

With a 2300-year history, TTM is an independent and comprehensive system of treatment, shaped by Tibetan plateau disease characteristics and their attended therapy practices. Rather than an offshoot of Chinese traditional medicine, TTM’s particular therapy outcomes are characterized by treating chronic disease, frequently-occurring disease and difficult diseases unique to Tibet. Based on TTM practice, TTM drugs are mainly made of natural herbs grown on the Tibetan plateau, most of which have lower prices than western drugs [9–14]. Furthermore, Tibetan medicine has a close relationship with Tibetan traditional culture [15]. TTM’s culture-attributes, its focus on unique therapies and its lower cost, mean that TTM is popular among local residents. Finally, TTM education now involves more student training in the field of medical ethics and doctor-patient interaction, which has led to TTM doctors’ displaying a better attitude towards patients, which has also contributed to the popularity of TTM.

During China’s pre-2009 health reform era, some townships did not have Tibetan medicine departments; and some counties did not have a county level Tibetan medicine hospital. To address these problems, both the national and local Tibetan governments have invested heavily into establishing a comprehensive Tibetan medicine service delivery system, including building Tibetan medicine departments in THC, building county level Tibetan medicine hospitals and improving service capacity of prefecture level Tibetan medicine hospitals [16]. Currently, most traditional Tibetan medicine hospitals operate at the prefecture level sharing health care provision with separate western medicine (WM) prefecture hospitals. Services are bifurcated, with WM hospitals mainly providing western medicine services, and TTM hospital’s providing traditional Tibetan medicine services.

On average, there are 237 health staff in prefecture WM hospitals compared with 71 health staff in prefecture TTM hospitals. Hospital staff in these WM and TTM hospitals were paid a fixed salary based on their professional grade, plus a floating salary that is determined by the total income of the hospital. For the fixed salary component, government is responsible for 85 % and the hospital itself pays the remaining 15 %. For all patients, the costs of health care are shared between the government and the individual, with the reimbursement rate for medically insured patients higher than for non-insured patients. TTM hospitals provide Tibetan medicine examination, treatment and drugs, such as color inspection, pulse taking, urinalysis, blood-letting therapy and external therapy [17–19]. China has a special health care delivery system. In terms of categories and levels of health care facilities, it includes hospitals and primary care facilities. But because there is no well-functioning gatekeeping and referral system, hospitals also provide primary care. Therefore, while providing specialist care for referral patients, one of the main functions of prefecture WM and TTM hospitals is to provide primary care for local residents.

Resource allocation between alternative types of primary health care providers poses the question of where China’s scarce health resources are best spent. The health reform agenda in Tibet has allocated significant primary health care funding to TTM, which raises the question of whether this allocation is an effective allocation of health resources. One measure of the best allocation of health care funding is patient assessments of the extent and quality of TTM versus WM health care services. Relying on a unique survey of patients’ relative assessment of two different prefecture-level Tibetan primary health care providers, this paper reports the first study to directly assess the performance of traditional Tibetan medicine versus western medical health care provision.

## Method

The Ethical Committee of Tibet Autonomous Regional Health and Family Planning Commission approved the study.

### Survey instrument

Success in achieving primary health care was measured against first contact and continuity; comprehensiveness (medical care); comprehensiveness (social care); first contact (access); coordination; family centeredness; community orientation; same doctor and stableness [7]. A validated Tibetan version of the Primary Care Assessment Tool (PCAT-T) was used for data collection. PCAT was developed by Johns Hopkins Primary Care Policy Center to measure the extent and quality of primary care services provided at a structure designated by patients as their main source of general care. Under very different health care systems outside the United States, modified PCATs have shown good cross-cultural adaptability for assessing primary care quality attributes from the patient’s viewpoint [20–24]. The validated PCAT-T has displayed good validity and reliability for primary care assessment in Tibet [7]. The details of PCAT-T were reported in one of our previous studies [8].

A four-point Likert-type scale was applied to measure certainty as to whether a service was received or not, ranging from “1” (“Definitely not”) to “4” (“Definitely”). A neutral response of “Not sure/ don’t remember”, assigned a median value of 2.5, measured the lack of knowledge about a characteristic, and also ensured consistency with methods used in PCAT studies in other countries [23]. We converted Likert scales to scores ranging from 25-100 by dividing the Likert scale by 4 and multiplying by 100. Means of item scores in the same scale yielded nine scale scores, and the primary care total score was the mean of these nine scale scores.

‘Regular health care provider’, was defined as the hospital where the respondents usually go to seek service or advice when they get sick. Other variables included socio-demographic variables (gender, age, education, occupation, income, marital status), health service utilization variables (patient visit frequency, inpatient status) and self-rated health status.

### Study design

Conducted on-site at the sampled health care structures in Tibet, our sample was based on face-to-face patient surveys. Using socioeconomic and geographic factors, a stratified, purposive sampling approach was used to select the two prefectures, Shigatse and Linzhi. Comprising 18 counties, Shigatse prefecture has 630,000 residents; an urban per capita disposable income of 14,700RMB; and the share of economic activity equally balanced between agriculture (24 percent) and industry (25 percent). Linzhi is a smaller, less populated (173,000 residents) and more industrial (industry 35 percent and agriculture 15 percent of economic activity) prefecture than Shigatse. In each prefecture, one prefecture WM hospital and one prefecture TTM hospital were selected. The sample sizes were estimated with reference to other similar studies that showed a sample size of 300 per group was needed for a significance level of 5 % with a power of 90 % for comparative analysis [25–28]. To compensate for expected missing data in some of the surveys, an additional 30 questionnaires were collected at each hospital, yielding 360 interviews at each of the two prefecture TTM and WM hospitals. In total, 720 surveys were administered.

Local health bureau officers were trained to conduct face-to-face interviews between September and October 2013. Patients visiting the family medicine, general internal medicine, and general obstetrics and gynecology departments, aged at least 18 years and who reported that the hospital was their regular health care provider were recruited for interview. Patients in the waiting room at each sample site were approached during a predetermined interval. The interval was calculated by dividing the expected patient population size by the required sample size. Each potential participant was given an explanation of the research purpose and asked for permission to participate in the interview. Patients were interviewed immediately after completing their health visit. While most of patients approached agreed to be interviewed, some patients refused, mainly due to their desire to travel immediately, sometimes involving long distances, to their home. Of the 720 questionnaires administered, 28 questionnaires were deleted due to missing data, leaving 692 completed questionnaires.

### Data analysis

Our analysis compares patient assessment of primary care quality attributes between WM hospitals and TTM hospitals in Tibet. First, chi-square tests were used to test for differences in socio-demographic, health service use and health status characteristics of the two groups. Next, t-tests were performed to compare the separate and total primary care attributes between WM hospitals and TTM hospitals. Finally, multiple linear regression analysis was conducted to examine the association of the health care setting with primary care attributes while controlling for socio-demographic, health service use and health status characteristics.

## Results

Patient socio-health demographics differed significantly between WM hospital and TTM hospitals, except for gender and employment status. Compared with WM hospitals, Table 1 shows that TTM hospitals had twice the percentage of old people (15.8 % versus 8.4 %), and patients with lower education levels (35.8 % with junior high school and above versus 66.0 %), lower income levels (46.9 % versus 26.5 % with annual household income below 30,000RMB) and patients more likely to be married (79.2 % versus 60.5 %). Also TTM hospitals had a higher proportion of patients visiting 4 times or more times (32.8 % versus 18.4 %) than WM hospitals.

**Table 1:** Comparison of patients’ socio-demographic characteristics and healthcare services use between WM hospital and TTM hospital

Characteristics	WM (%) (n = 332)	TTM (%) (n = 360)	*P* value
**Socio-demographic characteristics**			
Gender			0.956
Male	163(49.1)	176(48.9)	
Female	169(50.9)	184(51.1)	
Age			0.003
<60 years	304(91.6)	303(84.2)	
> = 60 years	28(8.4)	57(15.8)	
Education			<0.001
Below junior high school	113(34.0)	231(64.2)	
Junior high school and above	219(66.0)	129(35.8)	
Occupation			0.302
Employed	251(75.6)	284(78.9)	
Unemployed	81(24.4)	76(21.1)	
Income			<0.001
<=30000RMB	88(26.5)	169(46.9)	
>30000RMB	244(73.5)	191(53.1)	
Marital status			<0.001
unmarried	131(39.5)	75(20.8)	
married	201(60.5)	285(79.2)	
**Health service utilization**			
Number of PCP visits in the past year			<0.001
<=3	271(81.6)	242(67.2)	
> = 4	61(18.4)	118(32.8)	
Whether inpatient during the past year			0.085
Yes	59(17.8)	83(23.1)	
No	273(82.2)	277(76.9)	
**Health Status**			
Self-rated health			0.981
Healthy	218 (65.7)	230 (63.9)	
Unhealthy	114 (34.3)	130 (36.1)	

Note: WM = Western Medicine; TTM = Traditional Tibetan Medicine; *P*-value of chi-square test

Table 2 presents adult patient comparative assessments of primary care quality between WM hospitals and TTM hospitals. Overall, patient assessment of primary care performance in TTM hospitals scored 80.0 compared with 74.6 for WM hospitals. Patients in TTM hospitals reported significantly higher scores on a range of primary care assessments, including first contact and continuity, comprehensiveness (medical care), comprehensive (social care), coordination, family centeredness and same doctor. There was no significant difference in patient assessments of first contact (access) and community orientation between TTM and WM hospitals. Only in terms of the stableness score did WM hospitals outperform TTM hospitals.

**Table 2:** Comparison of primary care assessment score among adult patients between WM hospital and TTM hospital

Scales	WM Score Mean(SE)	TTM Score Mean(SE)	*P* value
First contact and continuity	83.64(0.78)	86.70(0.66)	0.003
Comprehensiveness (medical care)	76.93(1.04)	83.70(0.83)	<0.001
Comprehensiveness (social care)	77.63(0.90)	85.86(0.77)	<0.001
First contact (access)	58.35(1.16)	61.59(1.25)	0.059
Coordination	70.18(1.08)	84.53(0.89)	<0.001
Family Centeredness	80.13(0.85)	87.91(0.60)	<0.001
Community Orientation	66.72(1.13)	67.96(1.08)	0.430
Same doctor	70.59(1.41)	78.00(1.24)	<0.001
Stableness	51.90(1.23)	45.85(1.34)	0.001
Total	74.63(0.58)	80.00(0.44)	<0.001

Note: higher value indicates a more positive experience

WM = Western Medicine; TTM = Traditional Tibetan Medicine; SE = standard error

*P*-value of t test

Controlling for socio-demographic, health care utilization and health status characteristics, the regression results in Table 3 show that there was a significant association between health care settings and the primary care assessment total score. The average adjusted primary care score was 5 points higher at TTM hospitals (*P* < 0.001) than at WM hospitals. Among health care service utilization measures, number of primary care provider (PCP) visits in the past year was significantly associated with primary care quality. Patients who visited their PCP no more than 3 times in the past year reported higher score than patients who visited their PCP at least 4 times in the past year (*P* < 0.05). Healthy patients (*P* < 0.01) assessed the performance of PCP significantly higher than unhealthy patients. We found no significant association between socio-demographic factors (gender, age, income, marital status and occupation) and quality of care.

**Table 3:** Liner regression analysis on primary care assessment score

Dependent variable: Primary care achievement (total score)	B (95 % CI)	SE	P value
**Health care settings**			
WM	-	-	-
TTM	5.00(3.47-6.54)	0.78	<0.001
**Health care service utilization**			
Whether inpatient in the past year?			
Yes	-	-	-
No	0.32(-1.46-2.10)	0.91	0.726
Number of PCP visits in the past year			
<=3	-	-	-
> = 4	−1.89(-3.54--0.24)	0.84	0.025
**Health Status**			
Self-rated health			
Unhealthy	-	-	-
Healthy	2.68(0.89-4.47)	0.91	0.003
**Socio**-**demographic characteristics**			
Gender			
Male	-	-	-
Female	0.99(-0.43-2.41)	0.72	0.172
Age			
<60 years	-	-	-
> = 60 years	0.05(-2.24-2.34)	1.17	0.967
Income			
= < 30000RMB	-	-	-
>30000RMB	-0.07(-1.62-1.47)	0.79	0.926
Education			
Below junior high school	-	-	-
Junior high school and above	-1.42(-3.01-0.17)	0.81	0.080
Martial status			
Unmarried	-	-	-
Married	1.44(-0.16-3.05)	0.82	0.078
Occupation			
Employed	-	-	-
Unemployed	1.35(-0.40-3.11)	0.89	0.131

Note: WM = Western Medicine; TTM = Traditional Tibetan Medicine; SE = Standard Error; CI: Confidence interval

## Discussion

This study assesses patients’ evaluation of differences in primary care quality between TTM and WM hospitals. Our results showed that TTM hospitals had older patients, and patients with lower levels of education and income. One explanation is that most of the older patients and less-educated patients could only speak the Tibetan language and also sought lower medical costs. TTM was cheaper than WM, and physicians in TTM hospitals spoke the Tibetan language (as well as Mandarin), while physicians in WM hospitals usually only spoke Mandarin. The higher visit frequencies by patients using TTM hospitals is explained by TTM patients undertaking more frequent health care visits due to the chronic and comprehensive characteristics of TTM treatment, which usually included several periods of treatment [9–14].

TTM medicine is characterized by a long history, with a focus on Tibetan-specific diseases and reliance on Tibet’s natural medical ingredients for treatment. Our data show that TTM hospitals had a higher primary care assessment total score, especially on the scale of coordination, than WM hospitals. The higher score for coordination suggests that doctors in TTM hospitals pay more attention to following up their patients’ treatment outcomes. This can be explained by Tibetan medicine’s chronic and comprehensive treatment characteristics. TTM hospitals scored lower on the scales of stableness than WM hospitals. In spite of substantial investments in TTM hospitals, WM hospitals had a superior environment (building and sanitary condition) and better equipment and medical techniques. While constrained by the lack of Tibetan-speaking physicians in WM hospitals, TTM patients were more likely to seek treatment in WM hospitals than WM hospital patients were to seek treatment in TTM hospitals.

In Table 3, factors positively associated with better primary care quality among all respondents included having few hospital visits during the past year and self-rated good health status. Since patients who have less health service utilization are likely to be healthy, they tend to give positive rating of their hospital experience [29]. Importantly, the regression coefficient for patient’s socio-demographic factors, including age, occupation, education, income and gender, were insignificant in the study. Overall, patients rated TTM hospitals higher than WM hospitals at the prefecture level.

There are several limitations and areas for future research in this study. First, a self-reported survey was used to assess patient experiences. Since we cannot get technical quality information through patient survey approaches, self-report is the only way that people’s actual experiences can be assessed. Second, our results apply to prefecture level hospitals, but the Tibetan medicine delivery system also involves county and town level services. Similar studies of primary health care provision are required at county level and town level. For example, primary health care performs a gatekeeper role for the whole health system, managing referrals to specialist care and hospitals. Tibetan hospitals play both roles, gatekeeper and advanced care. Combining primary and tertiary health care within a single entity involves a trade-off between uncontrolled and inappropriate access to hospital services versus waste related to different organizations delivering different types of health care. By assessing the differences between TTM at prefecture level, county and township level, insights could be gained into the relative efficiency of Tibet’s different TTM health care providers. Third, this study only measures patients’ experience of care rather than the outcomes of primary care service. Further study is needed to examine how primary care attributes are related to actual health outcomes. Such a study can help us identify the main health care attributes, which are most closely related to outcomes in Tibetan context, so that limited resources can be used to focus on these areas. Fourth, the generalizability of this study result may be limited. In this study, only two TTM hospitals and two WM hospitals were involved. A larger study with a bigger number of hospitals will be needed in the future.

## Conclusion

In conclusion, our study demonstrated that TTM patients reported better primary care experiences than patients using WM hospitals. This pilot assessment provided evidence that the government’s investment in traditional Tibetan medicine was effective in Tibet. Future government policies should be developed to further build the capacity of Traditional Tibetan Medicine system in Tibet.

## Abbreviations

PCAT: Primary Care Assessment Tool
PCAT-T: Primary Care Assessment Tool-Tibetan version
TTM: Traditional Tibetan Medicine
WM: Western Medicine
